# Supplementary material for: DspA/E-Triggered Non-Host Resistance against E. amylovora Depends on the Arabidopsis GLYCOLATE OXIDASE 2 Gene
Source: Int J Mol Sci. 2022 Apr 11;23(8):4224. doi: 10.3390/ijms23084224 (PMC9029980; doi:10.3390/ijms23084224)
Supplement: Supplementary file 1 [file ijms-23-04224-s001.zip › ijms-1641015-supplementary.pdf]

Table S1. Sequence of primers used in this study.

Primer name	**Sequence (5′- 3′)**
start gox2-2-F	GGAGATAGAACCATGGAGATCACTAACGTTACCGA
stop gox2-2-R	TCC ACC TCC GGA TCC TAT AAC CTG GGC AAA TGG C
end gox2-2-R	TCC ACC TCC GGA TCA TAT AAC CTG GGC AAA TGG C
AtGOX2-S27Ff	GATCTTCTGCACCAAATGCATAGTAGTCATATACCATCTTAG
AtGOX2-S27Fr	CTAAGATGGTATATGACTACTATGCATTTGGTGCAGAAGATC
AtGOX2-A96Vf	CTACGGCTAGAGCTGTGTCTGCTGCTGGAAC
AtGOX2-A96Vr	GTTCCAGCAGCAGACACAGCTCTAGCCGTAG
T7	TAATACGACTCACTATA
T7-term	GCTAGTTATTGCTCAGCGG

(a) 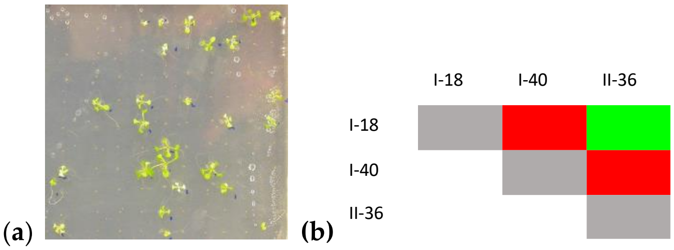 (b)

Figure S1. Segregation analysis of DspA/E suppressor mutants. (a) F2 progeny of I-18-1 x 13-1-2 sown on 5 µM estradiol. 27% of seedlings were resistant to 5 µM estradiol while 73% were sensitive to estradiol. **(b)** determination of complementation groups of DspA/E suppressor mutants. Putative suppressor mutants were crossed and seeds of the F1 progeny were sown on 10 nM estradiol to determine whether the putative mutations were functionally complementary or not. Germination of F1 on estradiol (green) indicates an absence of complementation and thus that the two mutations belong to the same complementation group. Lack of germination of F1 progeny (red) indicates that the mutant do not belong to the same complementation group.

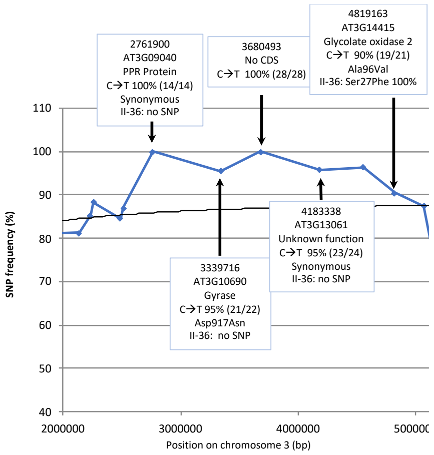

Figure S2. Region of chromosome 3 with common SNPs in suppressor mutants. SNPs present in I-18 and II-36 suppressors. Percentage of SNPs in chromosome 3. SNPs found in the two suppressor mutants in the 5 genes located in the high % SNPs region of chromosome 3. CDS: coding sequence.

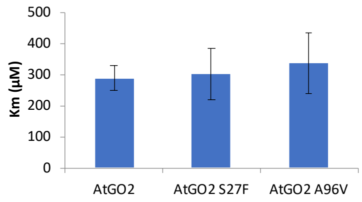

Figure S3. I-18 and II-36 point mutations do not affect Km glycolate of GOX2.
